# Dr. William Whitney Kitchen

**Published:** 1912-12

**Authors:** 


Dr. William Whitney Kitchen, formerly of Buffalo, accidentally shot himself while cleaning a revolver, Oct. 17. About two years ago, he was appointed U. S. Consul at Teneriffe. The remains were brought to Buffalo and the funeral was held from the Scottish Rite Cathedral.

